# A self-reported cross-sectional study on the oral function and the quality of life in children with stunted growth

**DOI:** 10.3389/fped.2022.1019143

**Published:** 2023-01-05

**Authors:** Eunike Sianturi, Risti Saptarini Primarti, Arlette Suzy Setiawan

**Affiliations:** ^1^ Pediatric Dentistry Residency Program, Faculty of Dentistry, Universitas Padjadjaran, Bandung, Indonesia; ^2^ Department of Pediatric Dentistry, Faculty of Dentistry, Universitas Padjadjaran, Bandung, Indonesia

**Keywords:** stomatognathia, mastication, swallowing, speech, Oral Health-Related Quality of Life, stunting

## Abstract

**Background:**

Failure to catch up with growth in toddlerhood will stunt elementary school children, which continues to cognitive decline and oral motor coordination. Verbal motor skills play an essential role in the oral function of the stomatognathic system, which includes mastication, swallowing, and speech. Therefore, early attention to oral function disorders of the stomatognathic system can avoid complications in children's nutritional status and quality of life.

**Objective:**

The objective of this study was to analyze the correlation of oral function of the stomatognathic system with Oral Health-Related Quality of Life (OHRQoL) in stunting children.

**Methods:**

This cross-sectional study correlates with 58 children aged 7–12 years with a history of stunting in toddlerhood from the Pasir Jambu District, Bandung Regency. The oral function of the stomatognathic system was evaluated by Adapted Orofacial Myofunctional Assessment Protocol and OHRQoL with Child Oral Health Impact Profile Short Form (COHIP-SF 19).

**Results:**

The results were statistically analyzed using Spearman's rank correlation and Kendall’s coefficient of concordance correlation tests. The results showed that the research subjects had a significant relationship in each variable and the three variables of the oral function of the stomatognathic system (chewing, swallowing, and speech) with OHRQoL with a *p*-value <0.05. The study concluded that the oral function of the stomatognathic system (chewing, swallowing, and speech) is related to OHRQoL in children with stunted growth.

## Introduction

According to the World Health Organization, stunting is defined as a condition of children aged 0–59 months with height for age below minus two standard deviations from the median standard to occur due to malnutrition for a long time in the first 1,000 days of life (1, 2). Stunted toddlers will manifest this stunting at school if they fail to catch up with their growth. The incidence of stunting in elementary school-aged children manifests stunting when they are toddlers because there is no improvement in the intake of macro- and micronutrients that are not needed in the long term. This nutritional problem almost occurs in all parts of Indonesia. Over one-third (36.1%) of primary-school-age children (7–12 years) in Indonesia are stunted due to chronic malnutrition (3). Stunted growth is a further consequence of a high number of low birth weight (LBW) infants and malnutrition in infancy and a lack of perfect growth improvement in the next period of their life. So it is not surprising that many school-age children are malnourished. Children with severe developmental delays can affect not only their reduced size but also their cognitive function. Most villages in West Java Province still experience a high prevalence of stunting, above 40%, including the Bandung Regency. Ten villages spread across eight subdistricts in Bandung Regency are prioritized for stunting management because of their high prevalence, namely, Rancatungku Village (Pameungpeuk District); Dampit, Narawita, and Tanjungwangi (Cicalengka District); Mekarlaksana (Cikancung District); Babakan (Cikarang District); Ciparay, Girimulya (Pacet District); Cihawuk (Paperari District); Karangtunggal (Paseh District); and Cibodas Village (Pasirjambu District) (2, 4, 5).

The impact of stunting is also associated with impaired brain development processes in the short term that affect cognitive abilities. Children who experience growth stunting will experience obstacles in their cognitive and motor development, affecting their productivity as adults. Stunting can affect cognitive decline and oral motor coordination (2, 6). Oral motor skills play an essential role in the oral function of the stomatognathic system (chewing, swallowing, and speech). The structure and function of the stomatognathic system usually develop according to the child's age. The formation of this system starts at 7 weeks of pregnancy until before the child is 6 years old (7).

Malnutrition can impair oral function affecting physical, neurological, motor, and social development. The process of mastication is a complex rhythmic activity that requires the free coordination of neuromuscular structures. Ikebe et al. reported that underweight individuals experience decreased muscle strength caused by energy deficiency (8). Poor nutrition causes changes in masticatory patterns and disrupted neurocranial formation and development. In addition, difficulty in chewing causes changes in preference for foods where children prefer soft foods to chew, which increases the risk of malnutrition and tooth decay (9–11).

Early attention to oral function disorders of the stomatognathic system can avoid complications in children's nutritional status and quality of life. Therefore, identifying, evaluating, and treating oral health and nutrition problems are vital in improving essential health and quality of life. However, research on the quality of life related to the oral function of the stomatognathic system is still limited (12–15). This study aimed to determine, analyze, and evaluate the relationship between the oral function of the stomatognathic system (chewing, swallowing, and speech) and the Oral Health-Related Quality of Life (OHRQoL) in stunted children. It is hypothesized that oral function correlates with the oral health quality of life of children with stunted growth.

## Material and method

### Design and sample

A school-based cross-sectional survey design was chosen to be appropriate for this study.

Determination of research locations was based on Bandung Regency Statistical Data that included eight subdistricts prioritized for handling stunting in 2019 (16); one of them is the Pasir Jambu subdistrict which was chosen randomly. The population of stunted children in these eight subdistricts is 249. At the same time, in Pasir Jambu, 58 boys and girls aged 7–12 years old had been listed in the Pasir Jambu Health Center to be diagnosed with growth stunting by their general practitioners or pediatricians during their preschool age (proven by data provided by Pasir Jambu Health Center). The recruitment of subjects was from January to March 2019. The minimum number of subjects required based on Slovin's formula (2% margin of error and 95% CI) (17, 18) leads to a minimum of 57 children. The nonprobability accidental sampling technique with inclusion criteria showed that the child can eat white bread (because the test will use white bread). Children with disabilities, such as physical disabilities and systemic diseases, were excluded.

### Instruments and protocols

This study assessed the oral function of the stomatognathic systems as mastication, chewing, swallowing, and speech activities. In addition, the masticatory function was assessed based on the child's type, method, and speed when chewing white bread weighing 0.05 g. The examination was carried out on the children one by one by the main researcher (first author). The results were adjusted according to the Adapted Orofacial Myofunctional Assessment Protocol criteria (19):

*Assessment of masticatory type*: Children were instructed to chew bread as we observed; then, we checked mastication by placing both palms on the cheeks for masseter muscle activity on both sides when the child chewed. This function is assessed as alternative bilateral (adequate) with a score of 0 and unilateral (inadequate) with 1.
*Assessment of how to chew*: Children were instructed to chew bread, and the researcher observed whether the child chewed bread with or without lip closure (20, 21). A child's lips are closed, with the upper and lower lips meeting, and the teeth are not visible when chewing bread. This function was assessed as the presence of lip closure (adequate) with a score of 0 and no lip closure (inadequate) with a score of 1.
*Assessment of chewing speed*: Children were instructed to chew bread, and the researcher measured the length of time the child chewed before swallowing bread using a stopwatch; then, the time was recorded by the researcher. This function is scored normal with a score of 0 if the child's mastication rate = 10 s, low with a score of 1 if the child's mastication rate is <10 s, and high with a score of 2 if the child's mastication rate is >10 s. The data obtained are on an interval scale.

Swallowing function was assessed when the child swallowed plain bread and mineral water. Researchers observed the presence or absence of the following activities when swallowing: sticking the tongue forward; moving the head up, down, right, and left; food is still in the mouth.; and lip and chin movement. The swallowing function is assessed as adequate with a score of 0 if the child does not show all of the above activities and inadequate with a score of 1 if the child shows one or more of the above activities.

The researcher assessed speech function by listening to the child when speaking spontaneously, mentioning pictures phonetically, instructing the child to say numbers from 1 to 20 and saying words, for example, “ring,” “book,” “ink,” and “photo” (Table 1). The function is assessed asadequate with a score of 0 if the child does not produce a distinct sound and inadequate with a score of 1 if the child produces a different sound.

**Table 1:** Tests for articulation disorders based on the Adapted Orofacial Myofunctional Assessment Protocol (19, 22).

Test	Example
Omission (phoneme omission)	Ool for pool
Substitution (phoneme replacement)	Thun for sun
Distortion (phoneme confusion)	Ing for ink
Inaccuracy (addition of phonemes/additions)	Phorto for photo

Children's quality of life was assessed using the Child Oral Health Impact Profile Short Form (COHIP-SF) Questionnaire consisting of 19 questions that were asked and recorded by the researcher directly to the child. Then, the answers were given based on the level of frequency, namely, a score of 0 if always (every day), a score of 1 if often (three times a week), a score of 2 if sometimes (once a week), a score of 3 if rarely (once a month), and a score of 4 if never. Total score = 0–76. Children's quality of life was assessed as good with a total score of >57, moderate with a score of 46–57, and poor with a total score of <46. The data were obtained on an ordinal scale.

The Indonesian version of the COHIP-SF Questionnaire has been retranslated into Indonesian from its original language (English) by the Universitas Padjadjaran Language Center. Children were asked to provide answers to a questionnaire that was asked directly by the researcher like how often they experienced oral health impacts over the last 3-month period. Only one examiner carried out all examinations.

The Indonesian version of COHIP-SF has been successful and published in a journal to be used as an OHRQoL instrument for school-age children in Indonesia. Internal consistency, test–retest reliability, discriminant validity, and convergent validity of the Indonesian version of COHIP-SF have been proven. The internal consistency and reliability of the COHIP-SF test–retest are perfect, with a Chronbach's alpha value of 0.83 and an intraclass correlation coefficient (ICC) of 0.81 (23–27).

### Data analysis

The type of research conducted on stunting children who meet the inclusion and exclusion requirements is a correlative type of research. Descriptive analysis in this study was used to describe the oral function of the stomatognathic system (chewing, swallowing, and speech) in stunted children. In addition, correlative analysis was carried out at the end of the study to determine, analyze, and evaluate the relationship between the oral function of the stomatognathic system (chewing, swallowing, and speech) and OHRQoL in stunted children.

Analysis was carried out using SPSS software with Spearman's rank correlation and Kendall’s coefficient of concordance correlation tests. Spearman’s rank correlation analysis results were used to determine, analyze, and evaluate the relationship between the oral function of the stomatognathic system (chewing, swallowing, and speech) with OHRQoL in stunted children for each variable. The Kendall coefficient of concordance analysis results were used to determine, analyze, and evaluate the relationship between the oral function of the stomatognathic system (chewing, swallowing, and speech) and OHRQoL in stunted children for all variables.

### Ethical aspects

The Health Research Ethics Committee of Universitas Padjadjaran approved this study (139/UN6.KEP/EC/2020). Signed informed consent was obtained from the children's parent or guardian before the study.

## Result

**CHART 1.**
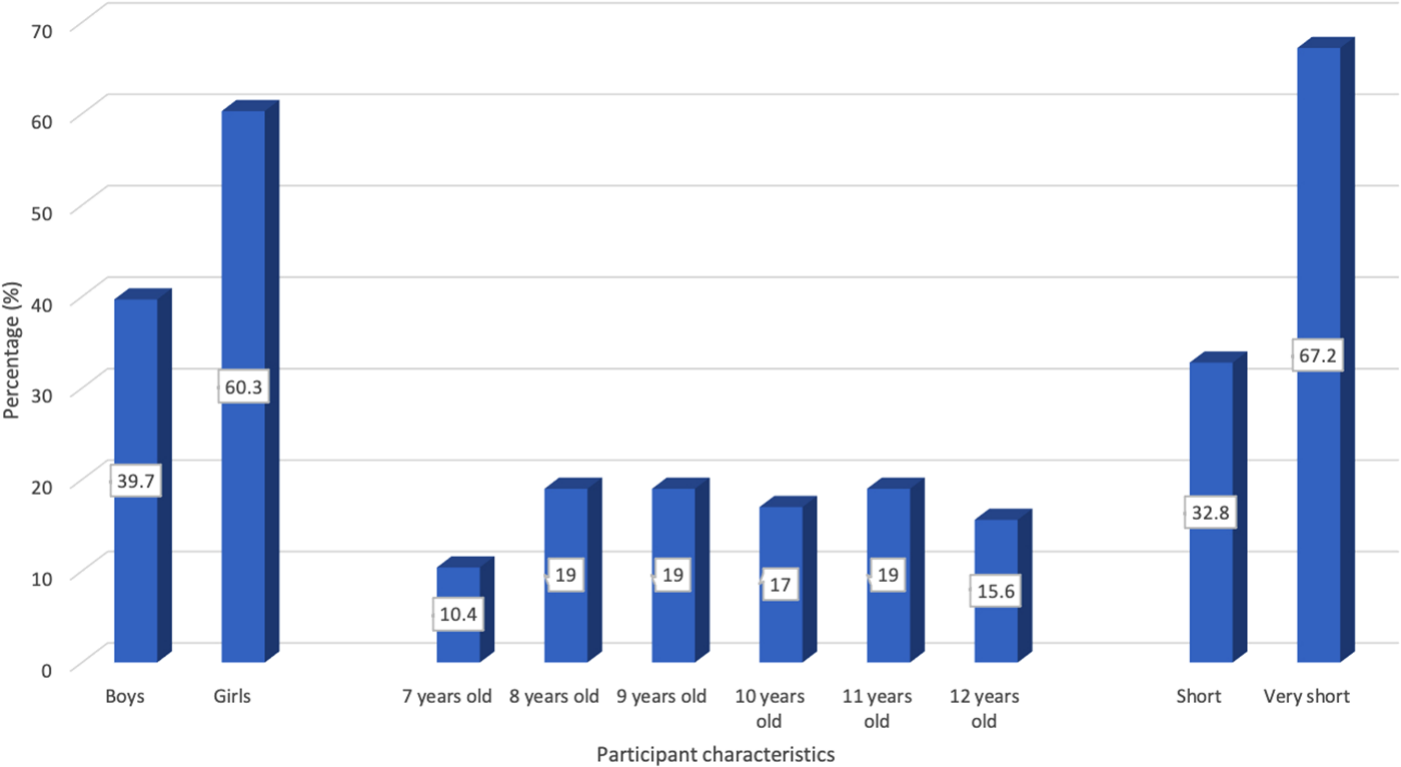
Characteristics of subjects (*n* = 58 children).

The study was carried out in a public elementary school in Puskesmas Pasir Jambu, Bandung Regency. The distribution of subjects based on stunting classification included 19 children with short stature (32.76%) and 39 with very short stature (67.24%). The characteristics of the research subjects above are shown in Chart 1.

Table 2 shows the study subjects’ examination results and assessment of stomatognathic function (chewing, swallowing, and speech). The masticatory function was seen unilaterally with a high chewing rate in all (100%) subjects, both in the very short groups. Lip closing mode during chewing showed that most subjects chewed without closing their lips (73.7% in the short group and 94.9% in the very short group). The function of swallowing food in Table 2 shows that the majority of subjects had inadequate activity in the anterior-projecting tongue position, atypical head, and static food (73.7%, 68.4%, and 63.2% of short children, respectively, while 94.9%, 92.3%, and 89.7% of very short subjects). Most of the periorbicularis oris muscle activity was adequate for both short subjects (89.5%) and very short subjects (71.8%). Mentalis muscle activity and lower lip interposition showed similar results in both groups of subjects (nearly balanced, adequate–inadequate on short subjects, and inadequate majority on very short subjects). The fluid swallowing function test results showed similar results to the swallowing function of food. Table 2 shows that inadequate activity was demonstrated for anterior tongue projection and atypical head in both groups of subjects. However, periorbicularis oris muscle activity and lower lip interposition showed adequate activity. Speech function tests are described in Table 2: activities that stand out as inadequate are spontaneous speech and phonetic pictures. For inadequate articulation activities, omission can be seen in the very short group and inaccuracy in the short group.

**Table 2:** Functions of mastication, swallowing, and speech.

Mastication function				
	Short stature [*n* (%)]		Very short [*n* (%)]	
Type		
Alternative Bilateral	—		—	
Unilateral	19 (100)		39 (100)	
Mode		
Lip closure	5 (26.3)		2 (5.1)	
Without lip closure	14 (73.7)		34 (94.9)	
Rate		
Normal	—		—	
Low	—		—	
High	19 (100)		39 (100)	
Food swallowing function				
Activity	Short		Very short	
	Adequate (%)	Inadequate (%)	Adequate (%)	Inadequate (%)
Tongue projection anteriorly	26.3	73.7	5.1	94.9
Atypical head	31.6	68.4	7.7	92.3
Static food	36.8	63.2	10.3	89.7
Periorbicularis muscle	89.5	10.5	71.8	28.2
Mentalis muscle	52.6	47.4	10.3	89.7
Low lip interposition	52.6	47.4	10.3	89.7
Liquid swallowing function				
Activity	Short		Very short	
	Adequate (%)	Inadequate (%)	Adequate (%)	Inadequate (%)
Tongue projection anteriorly	26.3	73.7	5.1	94.9
Atypical head	31.6	68.4	7.7	92.3
Periorbicularis muscle	94.7	5.3	87.2	12.8
Mentalis muscle	52.6	47.4	15.4	84.6
Low lip interposition	84.2	15.8	87.2	12.8

Speech function					
Test		Short		Very short	
		Adequate (%)	Inadequate (%)	Adequate (%)	Inadequate (%)
Spontaneous talk	52.6	47.4	25.6	74.4
Phonetic pictures	63.2	36.8	17.9	82.1
Numbers 1–20	84.2	15.8	87.2	12.8
Articulation	Omission	36.8	63.2	17.9	82.1
	Substitution	100	—	76.9	23.1
	Distortion	100	—	84.6	15.4
	Inaccurate	15.8	84.2	—	100

Children's quality of life related to oral health is presented in Table 3. In the domain of oral health conditions, most short and very short group subjects feel constantly disturbed by the presence of discoloration of the teeth and the condition of crowded teeth. The results showed varied results for the domain of health function conditions with no tendency for specific questions. The same is true for the domain of socioemotional health states. The results in Table 3 are then categorized into a lousy category with a mean score of 40.74 in the short group of children and 33.79 in very short children (Table 4).

**Table 3:** Oral Health-Related Quality of Life.

	Question	Always %	Often %	Sometimes %	Rarely %	Never %
Oral health condition domain						
Short	Pain in your teeth/toothache	—	42.1	57.9	—	—
	Discoloration of your teeth or spots on your teeth	100	—	—	—	—
	Crowded teeth or gaps between your teeth	100	—	—	—	—
	Bad breath	-	5.3	78.9	15.8	—
	Gums bleeding	-	10.5	42.1	47.4	—
Very short	Pain in your teeth/toothache	-	69.2	30.8	—	—
	Discoloration of your teeth or spots on your teeth	100	—	—	—	—
	Crowded teeth or gaps between your teeth	94.9	—	—	—	5.1
	Bad breath	—	7.7	82.1	10.3	—
	Gums bleeding	—	5.1	84.6	10.3	—
Health function condition domain						
Short	Trouble eating the food you want	—	—	78.9	21.1	—
	Having trouble sleeping	—	—	31.6	68.4	—
	Having trouble pronouncing certain words	—	—	52.6	47.4	—
	Having trouble keeping your teeth clean	—	—	15.8	52.6	31.6
Very Short	Trouble eating the food you want	—	5.1	87.2	7.7	—
	Having trouble sleeping	—	—	51.3	48.7	—
	Having trouble pronouncing certain words	—	2.6	82.1	15.4	—
	Having trouble keeping your teeth clean	—	—	28.3	71.8	—
Socioemotional health condition domain						
Short	Feeling unhappy or sad	—	—	57.9	42.1	—
	Feeling worried or anxious	—	—	31.6	68.4	—
	Avoid smiling or laughing with other children	—	5.3	57.9	36.8	—
	Feel that you look different	—	10.5	36.8	52.6	—
	Worrying about what other people think of you	—	15.8	52.6	31.6	—
	Bullied. bullied or teased by other children	—	—	15.8	73.7	10.5
	Skipping school	—	—	15.8	73.7	10.5
	Don’t want to talk	—	5.3	68.4	26.3	—
	Feel confident	—	—	52.6	47.4	—
	Feel attractive	—	—	52.6	47.4	—
Very Short	Feeling unhappy or sad	—	23.1	74.4	2.5	—
	Feeling worried or anxious	—	12.8	87.2	—	—
	Avoid smiling or laughing with other children	—	25.6	74.4	—	—
	Feel that you look different	—	56.4	43.6	—	—
	Worrying about what other people think of you	—	7.7	87.2	5.1	—
	Bullied. bullied or teased by other children	—	53.8	41.1	5.1	—
	Skipping school	—	2.6	69.2	25.6	2.6
	Don’t want to talk	—	5.1	84.6	10.3	—
	Feel confident	—	2.6	92.3	5.1	—
	Feeling attractive	—	2.6	92.3	5.1	—

OHRQoL, Oral Health-Related Quality of Life.

**Table 4:** OHRQoL in stunting children.

OHRQoL	Stunting (*n* = 58)	
	Short (*n* = 19)	Very short (*n* = 39)
Good (>57)	—	—
Fair (46–57)	—	—
Poor (<46)	Ⴟ = 40.74	Ⴟ = 33.79

OHRQoL, Oral Health-Related Quality of Life.

The relationship between OHRQoL and each variable of the oral function of the stomatognathic system in Table 5 was tested by Spearman’s rank analysis showing that there is a significant relationship between children's quality of life and masticatory function (correlation value: 5.60, *p*-value: 0.04) and swallowing (correlation value: 27.56, *p*-value: 0.00), as well as speaking (correlation value: 39.63, *p*-value: 0.00). Furthermore, the relationship between quality of life and the three variables of the oral function of the stomatognathic system tested with the Kendal coefficient of concordance showed significant results with a correlation value of 89.2, *p*-value <0.05 (Table 6).

**Table 5:** Relationship between OHRQoL with each variable in the oral function of the stomatognathic system (chewing, swallowing, speaking) and Spearman’s rank correlation.

Variable	rs	*t*	*p*-value		Correlation	Keterangan
OHRQoL—mastication	−0.24	−1.82	0.0369	*Sign*	5.60	Correlation
OHRQoL—swallowing	−0.52	−4.62	0.0000	*Sign*	27.56	Correlation
OHRQoL—speech	−0.63	−6.06	0.0000	*Sign*	39.63	Correlation

OHRQoL, Oral Health-Related Quality of Life.

**Table 6:** Relationship between OHRQoL and the three variables of the oral function of the stomatognathic system (chewing, swallowing, and speaking) with the Kendall coefficient of concordance.

Variable	Avg rank	Sum of ranks	*p*-value		Correlation	
OHRQoL	4	232	1.92E-33 (*p*-value < 0.05)	*Sign*	89.2	Correlation
Mastication	1.69	98				
Swallowing	2.91	168.5				
Speech	1.41	81.5				
Total	2.5	580				

OHRQoL, Oral Health-Related Quality of Life.

## Discussion

Slight closing angle and maximum closing speed are essential parameters that significantly affect masticatory performance. Children, by moving their tongue forward while chewing food, aim to exert efforts to improve their masticatory performance. Tongue movement contributes to the reduction of food by exerting shear forces on the food between the tongue and hard palate during the masticatory process, and the duration of the masticatory forces is significantly longer (28). The examination and assessment of the masticatory function of research subjects were inadequate. All children with short and very short stunting showed unilateral mastication, dominant mastication without lip closure and slow chewing speed in all short and very short stunted children.

Difficulty swallowing solids or liquids usually occurs in individuals with malnutrition and weight loss (29, 30). Multiple regression analysis revealed that maximum occlusal strength and the number of chewing cycles before swallowing were closely correlated with swallowing thresholds in children. These results suggest that the increase in the swallowing threshold is facilitated by the instantaneous strength of the masticatory muscles (including the maximum occlusal force) and the continuous activity of the masticatory muscles (31). Humbert et al. revealed that swallowing is associated with cognitive and motor processes in the brain. During swallowing, cognitively impaired patients show lower activation of the primary somatosensory and motor cortex (32). This supports the results that the function of swallowing food and drinks is inadequate in stunted children.

Perani et al. showed that neural networks for language acquisition are formed prenatally (33). The cross-sectional study by Chattopadhyay et al. showed that lower z-scores for height, weight, and head circumference were associated with a higher incidence of delayed or impaired language development (34). The short-term effects of malnutrition on child development are apathy, speech disorders, and other developmental disorders. Meanwhile, the long-term impact is decreased intellectual intelligence scores, cognitive development, sensory integration disorders, attention deficits, low self-confidence, and decreased academic performance in school (35). The study by Jimoh et al. showed a relationship between the development of nutritional status with low child weight and an increased risk of hearing delays, language domains, and interactive social. In addition, chronic malnutrition causes delays in the maturation of the auditory pathways that affect central and peripheral hearing, so that the child may have difficulty with spoken and written language (36). This study shows that all speech stunting children exhibit one or more of the above activities, which are used as parameters of inadequate speech function.

This study shows that malnutrition significantly affects all quality of life, including physical, emotional, and social functions. Nutritional status is one of the factors that affect the quality of life of children. Oral health status is closely related to quality of life (37–39). Research conducted by Wary also found that children with good nutritional status have a better quality of life than children with low nutritional status (40). Keshavarzi et al. stated that respondents with poor nutrition or at risk of malnutrition had a significantly worse quality of life than respondents with good nutritional status (41). Similarly, Istutiningrum, who examined the relationship between nutritional status and quality of life, showed a significant relationship between nutritional status and quality of life. Malnutrition influences all domains of children's quality of life, especially physical, emotional, and social functioning (42). Oral health conditions, dietary practices, nutritional status, and general health status are interrelated factors. Nutrition promotes the development and maintenance of healthy oral health. Malnutrition adversely affects the oral structure (5, 43).

The mean total OHRQoL score in this study in both groups showed a poor outcome. The research of Karamoy et al. on 321 school children aged 12 years living in Bekasi City and North Minahasa showed a significant relationship between dental caries status, dental and oral hygiene, and children's quality of life status using the COHIP-SF 19 questionnaire. This means that for children who have dental health problems, namely, the high number of dental caries and poor dental and oral hygiene status, then along with that, the quality of life of the child will decrease or be below (25).

Oral and dental health is an integral part of body health. A healthy body cannot be separated from having a healthy oral cavity and teeth, which affect a person's quality of life to chew, eat, swallow, and speak. The study by Husain et al. on 123 samples of stunted children in Enrekang Regency revealed a description of the quality of life related to dental and oral health (44) Growth retardation and retardation are associated with untreated dental caries and delayed eruption of permanent teeth in children in Cambodia, Indonesia, and Laos. Dimaisip-Nabuab et al. found that oral health could play an essential role in children's general growth and development (45). Difficulties in the development of oral function can lead to complications of malnutrition, as well as impaired physical and social development of children (7).

The limitation of the study is that *it* was conducted on subjects in one of the areas that became the locus of stunting*,* so it cannot be generalized *to* all areas in the Bandung Regency. Subjects were also not examined for conditions of the oral cavity that could affect oral function, such as the depth and shape of the palate. In addition, bread is not the type of food encountered by children in this location every day, so the results may be different from the children who are used to eating white bread. Therefore, further research is needed by involving other stunting locations in one city so that more complete results can be seen. Observation by a pediatric dentist is suggested to rule out some difficulties in chewing and even speaking.

## Conclusion

Oral stomatognathic system function (chewing, swallowing, and speech) is related to OHRQoL in stunted growth children. Therefore, this research shows that growth stunting can be managed from an early age.

## Data Availability

The original contributions presented in the study are included in the article/Supplementary Material, further inquiries can be directed to the corresponding author.
